# Biomarkers of early chronic obstructive pulmonary disease (COPD) in smokers and former smokers. Protocol of a longitudinal study

**DOI:** 10.1186/s40169-016-0086-5

**Published:** 2016-03-07

**Authors:** Mikael Truedsson, Johan Malm, K. Barbara Sahlin, May Bugge, Elisabet Wieslander, Magnus Dahlbäck, Roger Appelqvist, Thomas E. Fehniger, György Marko-Varga

**Affiliations:** Örestadskliniken, Eddagatan 4, 217 67 Malmö, Sweden; Section for Clinical Chemistry, Department of Translational Medicine, Lund University, Skåne University Hospital Malmö, 205 02 Malmö, Sweden; Centre of Excellence in Biological and Medical Mass Spectrometry, Biomedical Centre D13, Lund University, 221 84 Lund, Sweden; Clinical Protein Science and Imaging, Biomedical Centre, Department of Biomedical Engineering, BMC D13, Lund University, 221 84 Lund, Sweden; First Department of Surgery, Tokyo Medical University, 6-7-1 Nishishinjiku, Shinjiku-ku, Tokyo, 160-0023 Japan

**Keywords:** COPD, Clinical study, Biomarkers, Proteomics, Biobanking

## Abstract

**Background:**

Chronic obstructive pulmonary disease (COPD) is an irreversible disease, diagnosed predominantly in smokers. COPD is currently the third leading cause of death worldwide. Far more than 15 % of smokers get COPD: in fact, most develop some amount of pulmonary impairment. Smoking-related COPD is associated with both acute exacerbations and is closely correlated to comorbidities, such as cardiovascular disease and lung cancer. The objective of our study (KOL-Örestad) is to identify biomarkers in smokers and ex-smokers, with early signs of COPD, and compare these biomarkers with those of non-smokers and healthy smokers/ex-smokers. The participants in the study are recruited from Örestadskliniken, a primary health care clinic in Malmö, Sweden.

**Methods:**

Two hundred smokers and ex-smokers diagnosed with COPD with airflow restriction according to GOLD stages 1–4 will be included and compared with 50 healthy never-smokers, and 50 healthy smokers/ex-smokers without airflow restriction (total n = 300). The age distribution is 35–80 years. The participants undergo a health examination including medical history, smoking history, lung function measurements, and respond to a “Quality of Life” questionnaire. Blood samples are drawn every 6 months during a period of 5 years. Additional blood sample collection is performed if participants are experiencing an exacerbation. The blood fractions will be analyzed by standard clinical chemistry assays and by proteomics utilizing mass spectrometry platforms. Optimal sample integrity is ensured by rapid handling with robotic biobank processing followed by storage at −80 °C. The study has been approved by the Regional Ethical Review Board in Lund (http://epn.se/en), (Approval number: DNR 2013/480), and registered at the NIH clinical trial registry (http://clinicaltrials.gov).

**Results and discussion:**

Currently, 220 subjects are enrolled in the study.

**Conclusions and future directions:**

The study design will enable discovery of new biomarkers by using novel mass spectrometric techniques that define early changes of COPD. Such panels of novel biomarkers may be able to distinguish COPD from closely related diseases, co-morbidities, and contribute to an increased understanding of these diseases.

**Graphical abstract:**
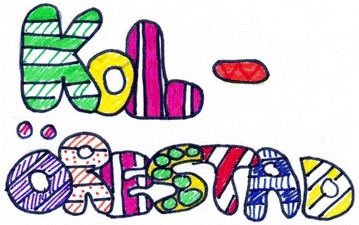
KOL-Örestad Study

## Background

Chronic obstructive pulmonary disease (COPD) is an irreversible disease, diagnosed predominantly in smokers. COPD is currently the third leading cause of death worldwide [1, 2]. It is a heterogeneous inflammatory disease characterized by different phenotypes, such as airflow limitation that is not fully reversible, chronic sputum production (chronic bronchitis) and destruction of parenchymal lung tissue (emphysema) [2]. Active smoking is the main risk factor for developing COPD. Far more than 15 % of smokers get COPD: in fact, most develop some amount of pulmonary impairment [3]. Smoking-related COPD is associated with acute exacerbations and is closely correlated to comorbidities, such as cardiovascular disease and lung cancer [2, 4]. The number of inflammatory cells and mediators is increased in the lungs and can be detected in bronchoalveolar lavage, biopsies and blood [5]. The pathophysiology of these systemic manifestations is unclear; however, several attempts to study biomarkers closely associated with COPD phenotypes have been performed, both in cross-sectional and longitudinal studies. Standardized methodology and strict quality control, replication of data in different cohorts, as well as robust and cost-effective clinical tests are needed [6]. Significant differences in biomarkers when comparing patients with COPD with former or non-smoking controls have been described [7]. Some biomarkers showed large variability within the three months study period. Plasma fibrinogen was the most ‘reliable’ analyte and was elevated, along with plasma CRP, in COPD patients experiencing exacerbations compared to COPD patients without exacerbations.

However, expiratory volume in one second (FEV_1_) is the most widely used parameter to evaluate the disease severity with increasing airflow limitations [2, 8]. The ratio of FEV_1_ to forced vital capacity (FVC) is measured with spirometry and is currently most frequently used to classify the disease state of COPD according to the Global initiative for Obstructive Lung Disease (GOLD) stages 1–4 [9]. Yet, FEV_1_ correlates poorly to clinical symptoms in early stages of COPD, but slightly better in later stages of the disease [2]. A study from Evaluation of COPD Longitudinally to Identify Predictive Surrogate Endpoints (ECLIPSE) found that the degree of airflow limitation does not reflect the heterogeneity of COPD. Additionally, a substantial part of the COPD patients, even with severe airflow obstruction, did not complain of any symptoms [10]. Furthermore, there are recent suggestions to classify structural changes in COPD using CT-imaging of morphological changes in pulmonary tissue tracts [11]. The most recent GOLD classification includes both symptoms and “Quality of Life” (QoL) in addition to lung function [12].

COPDGene is another front line study that was first to define both genetic factors implicated in COPD, as well as to investigate structural pulmonary changes with CT. The original cross-sectional study, which started 2008, has been extended with several follow-up studies to detect changes during disease progression [13].

Over the last decade, several studies have attempted to identify new biomarkers of COPD in blood, biomarkers both for diagnosing the disease as well as to predict exacerbations and to classify disease states [14, 15]. A number of studies on COPD plasma samples analyzed by mass spectrometry reported candidate protein biomarkers [16–18]. However, the current COPD studies available on patient blood samples analyzed by mass spectrometry are limited and there is still a lack of reliable biomarkers, a fact that complicates personalizing the care of patients with COPD.

The objective of this study (KOL-Örestad) is to identify circulating biomarkers in smokers and former smokers with early signs of COPD and compare these biomarkers with those of non-smokers and healthy smokers/ex-smokers. This could facilitate early diagnosis, evaluation of treatment outcome and more accurately classify patients with COPD. Participants are recruited from Örestadskliniken, a primary health care clinic in Malmö, Sweden. KOL-Örestad is the first COPD study to combine a primary health care, with a centralized large scale state hospital in a collaborative effort with academia.

## Methods

### Study outline

**Fig. 1:**
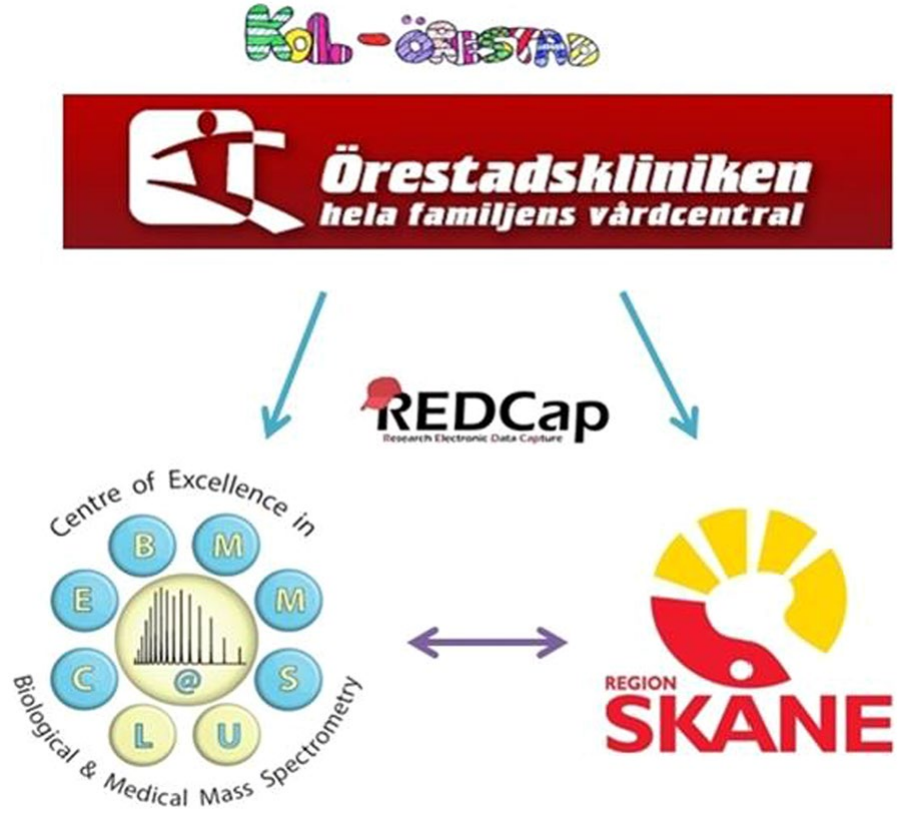
A schematic illustration of the partners within the COPD study: The primary health care clinic “Örestadskliniken”, the Skåne University Hospitals in Malmö and Lund “Region Skåne”, and the Centre of Excellence in Biological and Medical Mass Spectrometry (CEBMMS) at Lund University

This study is a close collaboration between a primary health care clinic (Örestadskliniken) in Malmö, the Skåne University Hospital in Malmö and Lund, and the Centre of Excellence in Biological and Medical Mass Spectrometry (CEBMMS, http://cebmms.lu.se) at Lund University, as depicted in Fig. 1, where the infrastructure of the study is illustrated. The study was initiated in 2014 and will continue for five years. The collaboration provides efficient blood sample access and high quality samples. The study has been approved by the Regional Ethical Review Board in Lund (http://epn.se/en), (Approval number: DNR 2013/480), and registered at the NIH clinical trial registry (http://clinicaltrials.gov).

Blood biomarkers from two hundred smokers and ex-smokers with diagnosed COPD according to GOLD stage 1–4 will be compared to those of a control group of 50 healthy never-smokers and 50 healthy smokers/ex-smokers (Table 1). Exclusion criteria include presence of a chronic inflammatory disease or treatment with steroids or any other immunomodulatory treatment that is unrelated to exacerbations of COPD. These conditions may otherwise interfere with the biomarkers measured in the study.

**Table 1:** Study participant distribution according to the GOLD standards

Study groups by pack years and air flow limitation	Pack years 1 pack year = 20 cigarettes a day in 1 year	Airflow limitation FEV_1_/FVC < 70 %
Healthy never smokers n = 50	0	No
Healthy smokers/ex-smokers n = 25	<20	No
At risk smokers/ex-smokers n = 25	>20	No
GOLD stage 1 smokers/ex-smokers n = 75	>20	FEV_1_ ≥ 80 %
GOLD stage 2 smokers/ex-smokers n = 75	>20	50 % ≤ FEV_1_ < 80 %
GOLD stage 3 smokers/ex-smokers n = 25	>20	30 % ≤ FEV_1_ < 50 %
GOLD stage 4 smokers/ex-smokers n = 25	>20	FEV_1_ < 30 %

The enrolled subjects will be divided in the following study groups. The total number of participants will be 300, including 50 healthy never smokers, 25 healthy smokers and ex-smokers, 25 at risk smokers and ex-smokers, 200 participants with GOLD stage 1–4 divided in 75 with GOLD stage 1 and 2 respectively (smokers and ex-smokers) and 25 with GOLD stage 3 and 4 respectively COPD participants (smokers and ex-smokers)

The participants will undergo health examinations including heart and lung, as well as blood pressure and spirometry. They will also fill out a QoL questionnaire regarding smoking habits, COPD symptoms, disease history and current medication. Blood samples are collected every six months, which are processed and stored as individual samples of plasma, buffy coat, red blood cells, serum and whole blood. Blood samples are collected in separate tubes (vacutainers) containing sodium heparin, sodium citrate or EDTA (ethylenediaminetetraacetic acid) as anticoagulants, or clot activator for serum separation. Separate plasma tubes are used to analyze a defined list of biomarkers at the Clinical Chemistry Laboratory at Skåne University Hospital in Malmö. Additional blood sample collection is performed when participants experience exacerbations.

**Fig. 2:**
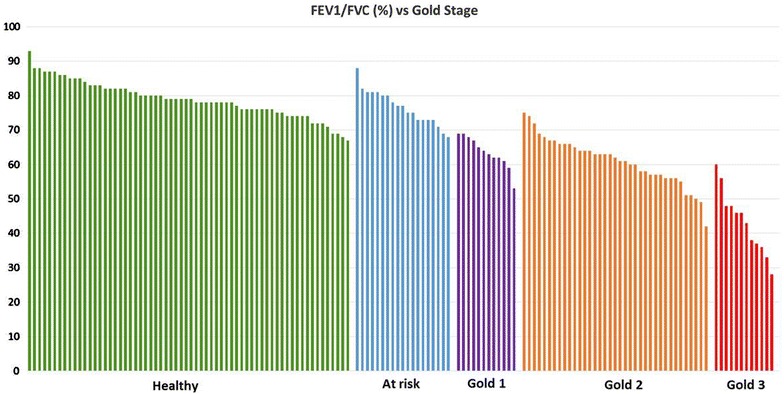
Lung function and GOLD stages of the participants currently enrolled in the study. The *y-axis* represents the FEV_1_/FVC ratio from the spirometry test and the *x-axis* shows patient classification in the following groups: healthy, at risk and COPD participants GOLD stage 1–3

All data are stored in a clinical database, using Research Electronic Data Capture (REDCap) as a data capture tool [19]. The blood samples will be stored at −80 °C in the biobank and subsequently be analyzed by liquid chromatography mass spectrometry (nano LC-MS/MS) techniques at CEBMMS, Lund University. The current status of the study recruitment and the participant distribution according to the GOLD standards are shown in Fig. 2. Currently, there are more participants in the healthy group and in GOLD stage 2, while participants in GOLD stage 4 are being recruited.

### Clinical samples and biobanking

The biobanking workflow has previously been developed at CEBMMS for rigorous standardization for large scale biobanking [20–23]. The blood samples are collected at the primary health care clinic and transported by a courier to the biobank at CEBMMS for aliquoting and storage. Four vacutainers (EDTA, citrate, heparin, serum) are centrifuged at 2000*g* for 10 min at room temperature in order to separate blood into three fractions: plasma, buffy coat and red blood cells. The automatic aliquoting of samples is performed by a robot (HAMILTON robot MicroLab Starlet, Hamilton, Bonaduz AG, Switzerland) that aliquots 70 μL of the sample per vial into half of the sample rack of a 384-well plate. Thus, blood from two subjects can be stored in parallel into one 384-well plate. The 1-D barcode on the vials arriving from the clinic are matched with another 1-D barcode on the 384-well plate. Each individual aliquot is subsequently labeled with a 2-D barcode encrypted for each patient and sample type to provide full traceability of the samples. The aliquot vials are then sealed with aluminum foil and punched individually. All plates are stored at −80 °C in a fully automated biobank storage unit (LICONIC freezer STT1k5 ULT, Liconic AG, Mauren, Liechtenstein) that is able to store clinical samples at a density of five million tubes [23]. This procedure, from sample collection to storage in the biobank, takes less than 2 h, which ensures high sample quality and safe handling of the samples. By producing multiple aliquots from the same sample, the freezing-and-thawing procedure is avoided, which may otherwise decrease sample quality. Each participant is assigned a unique identifier (Study ID) allowing all samples and data to be handled anonymously. The study ID can only be deciphered by clinicians at Örestadskliniken. For analysis, the aliquot vials can be retrieved via electronic command through the computerized biobanking system.

### Clinical database

The data acquired are stored in a database, KOL-Örestad database, including clinical patient data, blood pressure, spirometry, and a QoL questionnaire detailing smoking habits, COPD symptoms, and disease history. By use of the barcode or patient study ID, all clinical demographics, type of blood samples, and analyzed output data stored in the database, can be traced from each patient. REDCap from Vanderbilt University, TN, USA is selected as an electronic data capture tool for collection and managing the study data. REDCap provides a flexible and easily programmable interface especially adapted for clinical studies [19]. REDCap has user-specified input modules for a specific cluster of variables, e.g. modules on: QoL, blood sampling, spirometry, without restriction in time and numbers. User allowance can be adjusted for different users, e.g. clinicians and researchers. This ensures patient integrity and maximizes flexibility of the platform. All patient data that are handled outside the health care sector is encrypted and handled anonymously.

REDCap also functions as a planning instrument for clinicians, facilitating the planning of the number of patient follow ups each participant has completed. This gives the involved clinicians and researchers an excellent overview of all participants and their level of completion in the study.

**Fig. 3:**
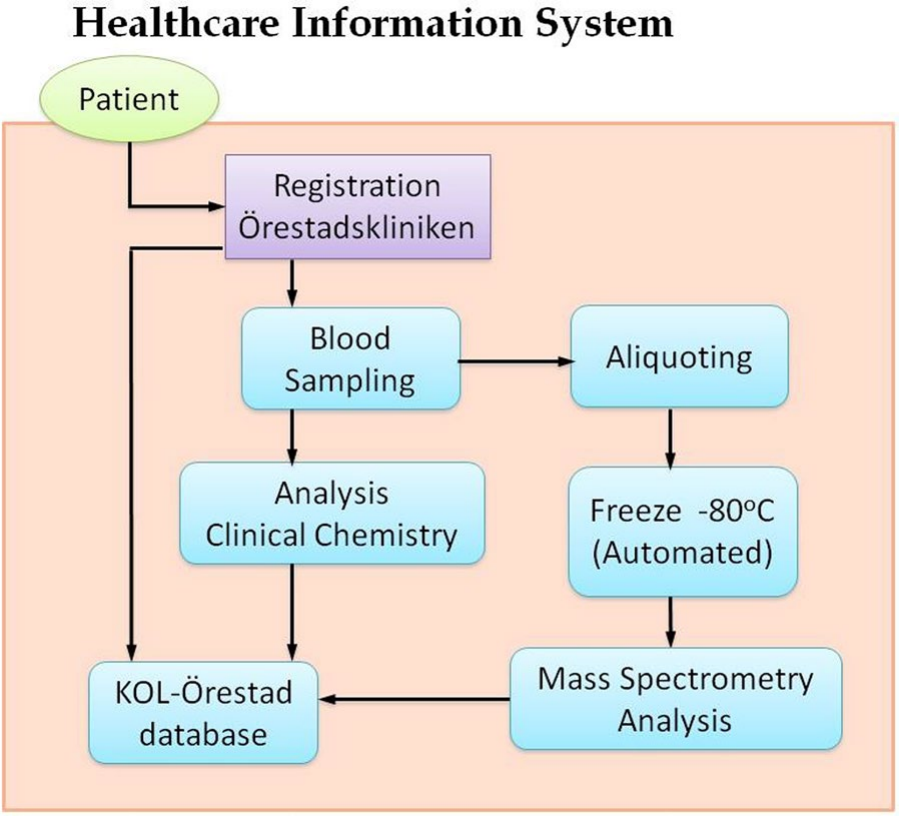
A demonstration of the workflow with Health Information System (HIS). The patient is registered at Örestadskliniken, the primary health care clinic. Blood samples are sent to Clinical Chemistry at the Skåne University Hospital in Malmö and to CEBMMS for analysis and storage in the biobank. These data, as well as clinical data from Örestadskliniken, are collected within the KOL-Örestad database

Health Information System (HIS) provides the foundation for decision-making and is comprised of four key functions: data generation, compilation, analysis and synthesis, and communication and use (Fig. 3). Data from the health care sector is collected within HIS as well as analyzed and controlled for quality, relevance, and timeliness [24].

### Data acquisition

The blood samples will be prepared for data acquisition by mass spectrometry by dilution, addition of chicken lysozyme, solubilization, reduction, alkylation, digestion with trypsin, spiking with heavy labeled peptide standards and centrifugation. More details of the preparations will be optimized and validated for a multiple reaction monitoring (MRM) workflow for the potential protein biomarkers from the collected blood samples, which will be analyzed by liquid chromatography mass spectrometry (nano LC-MS/MS). The confirmation of protein identities will be carried out based on a library obtained from Proteome database search (v 1.4, Thermo Scientific, San José, CA) and prior data dependent analysis (DDA) of pooled plasma samples from COPD patients and healthy participants.

The data input and logistical work flow is depicted in Fig. 3, followed by data output from resulting assay read-outs. Separate blood samples will be analyzed by standard clinical chemistry assays.

## Results and discussion

The aim of the study includes novel biomarker technology development on blood collections of patients with COPD and additional sample collections during exacerbations. COPD patients who also have cardiovascular diseases and lung cancer are included in the study, enabling cross over multi-factorial effects and co-morbidities to be investigated. Currently, 220 subjects are enrolled in the study. Because screening with spirometry is not regularly performed in the primary health care clinic on the aging population, new available tests would greatly benefit diagnostics. Biobanks are highly important for the introduction of personalized medicine in future health care [25, 26]. Preservation of clinical samples collected daily is an essential  undertaking, because each sample is unique and has the potential to facilitate future research and diagnostics.

Quantitative mass spectrometry provides an excellent method to identify biomarkers that can be used in personalized medicine. Mass spectrometry is very sensitive, which makes sample quality even more important. The present system generates aliquots of 70 μL in a 384-well plate using an automated sample processing platform. This minimizes the probability of methodological errors and improves sample quality by avoiding the use of previously thawed samples [23]. In our workflow, we have developed a method for safe handling of samples to maximize sample preservation, which is robust and well suited for clinical studies.

## Conclusion and future directions

In this study, the disease progression will be followed longitudinally for each patient. Novel disease biomarkers identified by proteomics (significantly up- or down-regulated due to disease state) will be related to measured clinical parameters. The study design will enable the discovery of new biomarkers that define early changes of COPD, distinct from markers of closely related diseases, such as cardiovascular disease and lung cancer. Such panels of novel biomarkers can contribute to an increased understanding of these diseases.

## Abbreviations

GOLD: global initiative for chronic obstructive lung disease
COPD: chronic obstructive pulmonary disease
QoL: quality of life
HIS: health information system
nano LC-MS/MS: liquid chromatography mass spectrometry
